# Identifying disease vector images in the Americas in the age of artificial intelligence

**DOI:** 10.1590/0037-8682-0291-2025

**Published:** 2025-11-03

**Authors:** Vinícius Lima de Miranda, José Fabrício de Carvalho Leal, Isadora Ribeiro de Carvalho Gomes, Taís Oliveira de Araújo, Rodrigo Gurgel-Gonçalves

**Affiliations:** 1 Universidade de Brasília, Faculdade de Medicina, Laboratório de Parasitologia Médica e Biologia de Vetores, Brasília, DF, Brasil.; 2 Fundação Oswaldo Cruz, Núcleo de Epidemiologia e Vigilância em Saúde, Programa de Residência Multiprofissional em Vigilância em Saúde, Brasília, DF, Brasil.

**Keywords:** Neglected tropical diseases Vectors, Public health, Automated identification

## Abstract

Digital technologies and artificial intelligence (AI) have become integral in many fields, including medicine. Neglected tropical diseases transmitted by vectors, such as arboviral diseases, spotted fever, Chagas disease, and leishmaniasis, pose a significant impact on public health, particularly in the Americas. Strengthening surveillance and control requires the use of digital technology to identify vectors. In this study, we explored how AI can aid in identifying vectors in the Americas and strengthen disease surveillance and control efforts. We reviewed the literature on the automated identification of triatomines, mosquitoes, sand flies, and ticks, focusing on advances in the Americas over the last 10 years, and provided a critical analysis of the automated identification systems for each group. Moreover, we analyzed the development stages of each study: image acquisition, image processing, algorithm training, algorithm testing, app development, app availability, and AI-based devices for vector identification and surveillance. Most studies have applied AI to identify mosquito species. The vector species databases were not diverse, and the most representative group was Triatominae, comprising 65 species (41% of all described species). Currently, approximately 30 algorithms are used for automated vector identification, with the most common being AlexNet, MobileNet, and ResNet. Most studies are in the algorithm training stage, and in the Americas, only one study has progressed to the development of applications or devices. These results highlight the potential of AI for identifying vectors in the Americas, supporting the use of automated visual identification systems as a promising approach to improve vector surveillance, while also promoting citizen science.

## INTRODUCTION

Digital technologies and artificial intelligence (AI) have become integral to many fields, including medicine^1^. The ability of machine learning algorithms to identify images plays a critical role in the surveillance of vector-borne diseases^2^. Neglected tropical diseases transmitted by vectors, such as arboviral diseases, spotted fever, Chagas disease, and leishmaniasis, pose a significant impact on public health, particularly in the Americas^3^ ^,^ ^4^. Strengthening surveillance and control efforts requires the use of digital technologies to identify vectors, such as mosquitoes, ticks, kissing bugs, and sand flies, as vector control remains a cornerstone of vector-borne disease prevention^5^.

Currently, vector identification services in the Americas rely on skilled professionals and specialized equipment, which are not always available. Strengthening vector surveillance could benefit from community participation, such as household notifications that support vector detection. In this context, automated visual identification systems offer a promising approach to improving surveillance while also fostering citizen science^6^. 

Over the past decade, scientists worldwide have made significant progress in developing digital technologies for vector identification^2^. However, there remains a notable gap in such advancements in the Americas, a region that harbors the highest diversity of vector species globally, including triatomines^7^. In this review, we explored how AI can aid in identifying vectors in the Americas and strengthen disease surveillance and control efforts. We reviewed the literature on the automated identification of triatomines, mosquitoes, sand flies, and ticks, focusing on advances in the Americas over the last 10 years, and provided a critical analysis of the automated identification systems for each group. Furthermore, we presented an overview of the current trends and future directions in this field.

We searched for literature containing the terms ‘automated identification,’ ‘mosquitoes,’ ‘ticks,’ ‘triatomines,’ or ‘sand flies.’ All relevant article types, such as original articles, reviews, commentaries, opinion pieces (from PubMed), grey literature, reports, and digital media-in English, Portuguese, and Spanish, were included. Additional materials identified through manual searches were also incorporated. To organize the findings, we compiled a table summarizing key information extracted from each study, including the taxonomic group, number of species analyzed, algorithms used, training hyperparameters, and accuracy. We also assessed the development stage of each study: image acquisition, image processing, algorithm training, algorithm testing, app development, app availability, and AI-based devices for vector identification and surveillance. The review is structured by taxonomic group, according to the amount of available scientific evidence.

## CULICIDAE (MOSQUITOES)

Mosquito-borne diseases represent a major public health concern, with more than half of the world’s population exposed to arboviruses such as dengue^8^. Recently, the application of AI in mosquito identification has advanced considerably, particularly through convolutional neural networks (CNNs). A PubMed search using the keywords ‘automated identification’ and ‘mosquito’ yielded at least 38 articles, nine of which originated from the Americas (Table 1). Most studies on automated mosquito identification have been conducted in Asia.

**TABLE 1:** Highest accuracies obtained by studies on automated identification of disease vector images considering taxonomic groups, number of taxa, algorithms, and training hyperparameters.

Group	Taxonomic level	Number of taxa	Algorithms	Highest accuracy (%)	Training hyperparameters	Author
Culicidae	Species	2	AlexNet	97	Learning rate = 0.001; epochs = 200; training: scratch with small dataset	Sanchez-Ortiz et al.^12^
Culicidae	Species	3	LeNet	76	Variable learning rate and epochs; optimizer: NAG, Adam, AdaGrad, SGD.	Motta et al.^14^
			AlexNet			
			GoogLeNet			
Culicidae	Species Genera	2	Perceptron	95	Learning rate = 0.5; training method: delta rule	Carrillo et al.^16^
Culicidae	Species	3	ResNet-50	97	Variable learning rates and epochs; batch size = 32-256; optimizer: RMSprop, Adam, AdaGrad, SGD.	Motta et al.^15^
			VGG-16			
			XceptionV3			
			DenseNet-201			
Culicidae	Species	67	Xception	97	Learning rate = 0.01; epochs = 60; optimizer = Ranger (RAdam + LookAhead); Batch size = 16.	Goodwin et al.^19^
Culicidae	Species	2	Dual-Source DNN	94	Learning rate = 0.00001; epochs = 1000; optimizer = RMSProp.	Arista-Jalife et al.^13^
			Shallow-NN			
			Slim-DNN			
			Robust-DNN			
Culicidae	Species	53	Xception	94	Variable learning rate. Monte Carlo simulation used for unknown species detection	Gupta et al.^20^
Culicidae	Species Genera	5	VectorBrain (EfficientNet-26)	94	Learning rate = 0.005; epochs = 100; optimizer: AdamW.	Li et al.^18^
Culicidae	Species	4	AlexNet	94	Learning rate = 0.001; epochs = 50	Araújo et al.^17^
Ixodidae	Species	3	CNN “TickIDNet”	88	Learning rate = 0.004; mini-batch size = 32; optimizer = SGD.	Justen et al.^30^
Ixodidae	Species	4	ResNet-50	80	Learning rate = 0.01; epochs = 50; batch size = 64; optimizer = Adam;	Omodior et al.^31^
			Custom CNN			
Ixodidae	Species	31	LeNet (TickPhone)	96	Epochs = 60; optimizer = support vector machine (SVM);	Xu et al.^32^
Ixodidae	Species	7	CNNs (Inception-ResNet)	92	Learning rate = 0.001; batch size = 64; optimizer = Adam.	Akbarian et al.^33^
Ixodidae	Species	3	VGG-16	99	Learning rate = 0.00001; batch size = 32; Adam10-fold cross-validation.	Luo et al.^34^
			ResNet-50			
			InceptionV3			
			DenseNet-121			
			MobileNetV2			
Ixodidae	Species	6	AlexNet	94	Learning rate = 0.001; number of epochs = 50; optimizer:Adam.	Gomes et al.^35^
			ResNet-50			
			MobileNetV2			
Triatominae	Species	51	Statistical Classifiers	88	Classification strategy = pairwise binary classifiers;	Gurgel-Gonçalves et al.^40^
Triatominae	Species	51	InceptionV3	83	Training steps = 2000-12000	Khalighifar et al.^41^
Triatominae	Species lineages	3	Multilayer	91	Learning rate = 0.1;	Cruz et al.^42^
			Perceptron Neural		Number of harmonics = 25;	
			Networks		number of PCA components = 30;	
Triatominae	Subfamily	51	VGG-16	99	Learning rate = 0.0001; epochs = 20;	Abdelghani et al.^43^
Triatominae	Species	51	VGG-16	97	Learning rate = 0.0003; weight decay = 0.002; epochs = 350; batch size = 64-128	Parsons & Banitaan^44^
			7-layer CNN			
Triatominae	Subfamily	2	ResNet-34	96	Learning rate = lr_find(); training steps = 50; training strategy = discriminative layer training;	Cochero et al.^45^
Triatominae	Genera	3	AlexNet	100	Learning rate = 0.15-0.001; epochs = 15	Miranda et al.^7^
			AdaBoost,			
			Gradient Boosting			
			Histogram-based			
			Gradient Boosting			
			Linear Discriminant Model			
Triatominae	Species	65	AlexNet	93	Learning rate = 0.001; epochs = 50	Miranda et al.^46^
Triatominae	Order Subfamily	3 2	AlexNet	96	Learning rate = 0.001; epochs = 100	Miranda & Gurgel-Gonçalves^47^
			MobileNetV2			
			ResNet-50			
Phlebotominae	Species	12	MobileNet	96	Batch normalization = enabled;	Cannet et al.^51^
	Genera	3	ResNet-20		SURF descriptors + Bag of Features (BoF) + SVM;	
	Subgenera	6	YOLOv2		dictionary size (BoF) = 4000 codewords;	
	Family	1	DarkNet		SVM kernel = polynomial	

In Korea, Kim et al.^9^ presented an automated system that detects mosquitoes using either a single image classifier (52% accuracy) or multiple deep-learning networks (84% accuracy). Okayasu et al.^10^ compared machine learning and CNN-based approaches using images of *Aedes*, *Culex*, and *Anopheles* in Japan. Models such as AlexNet, VGGNet, and ResNet achieved accuracy rates up to 95.5%, outperforming traditional data mining methods (82.4%). Similarly, Park et al.^11^ in South Korea tested VGG-16, ResNet-50, and SqueezeNet CNNs, achieving identification accuracies of 97%, 96%, and 90%, respectively, for eight species. Their findings demonstrated that fine-tuning and data augmentation substantially enhanced network performance. Heat-activation maps further showed that the networks focused on morphological features comparable to those employed by taxonomists, including leg structure and coloration patterns.

In the Americas, Sanchez-Ortiz et al.^12^ conducted a pioneering study on automated mosquito identification. They developed a larval mosquito image database and applied CNNs for classification. Arista-Jalife et al.^13^ effectively recognize larval samples with an accuracy of 94% and automatically cropped the larval region of interest to classify them as *Aedes*-positive or -negative without human intervention. Motta et al.^14^ used the LeNet, AlexNet, and GoogleNet networks to identify adult *Aedes aegypti*, *Aedes albopictus*, and *Culex quinquefasciatus* in the field, achieving 76.2% accuracy, which later improved to 93.5% after hyperparameter adjustments^15^.

Most studies aimed at automating the identification of mosquitoes have focused on distinguishing species belonging to the genera *Aedes*, *Culex,* and *Anopheles*; however, some studies have explored other mosquito vectors. Carrillo et al.^16^ achieved 95% accuracy in distinguishing *Limatus durhamii* from *Wyeomyia* spp. based on wing characteristics using a perceptron network. Araujo et al.^17^ applied AlexNet network to identify four wild mosquito species belonging to the genera *Sabethes*, *Haemagogus*, and *Aedes*, which are vectors of yellow fever, achieving >90% accuracy across different body regions. Li et al.^18^ developed VectorBrain, a lightweight deep-learning model capable of identifying *Anopheles* species, sex, and abdominal status from field sites in Africa and North America with high accuracy (Table 1). Goodwin et al.^19^ developed a CNN that achieved accuracies between 86% and 97% across 2,696 images of 67 species. Based on these models, Gupta et al.^20^ developed an IDX device, integrating high-resolution optics with computer vision algorithms, achieving 80-93% accuracy for 53 adult mosquito species. When tested on data from the United States and Papua New Guinea, the accuracies ranged from 55.3% to 93.6%.

Future studies in the Americas could leverage technologies already developed in the Old World, including algorithm optimization and robotics for vector identification. For instance, Jeyakodi et al.^21^ developed the mAedesID, an Android-based application using a customized CNN to classify *Ae. aegypti* and *Ae. albopictus*. The model was supported by techniques that reduced redundancies in the images and achieved an accuracy of 84.87%. Other studies have explored lighter models to avoid the need for large amounts of computational power. For example, Ong et al.^22^ used MobileNet and achieved an accuracy of 98.06% by using a model applicable to smartphones. Montalbo et al.^23^ used EfficientNet B0, which has low memory consumption (437 KB) and achieved 99% accuracy. Maruf^24^ proposed TransembleNet, an ensemble approach based on transfer learning, to classify mosquito vector species. This model combined InceptionV3, VGG-16, and ResNet-50, and outperformed previous methods with high accuracy and robust metrics. 

CNNs and digital sensors have transformed mosquito identification and surveillance systems. Smart traps integrated with computer vision and neural networks can accurately identify species, such as *Ae. aegypti* and *Cx. quinquefasciatus* ^25^. Other automated computer vision-based devices can capture and identify images of mosquitoes. A high-resolution optical system integrated with computer vision algorithms provides an accurate identification of adult mosquito species. The system acquires two distinctly oriented images of each specimen and immediately aggregates the data into a template report format^26^. They demonstrated the importance of refining algorithms and expanding datasets to cover more species and regions, which could enhance identification systems. Finally, AI-enabled mosquito surveillance using CNNs and robots designed to capture mosquitoes using multistage mosquito attractants is proposed. Semwal et al.^27^ developed the robot ‘Dragonfly,’ which captures mosquitoes and uses the YOLOV4 algorithm trained to identify *Ae. aegypti, Ae. albopictus*, and *Culex*. The ‘Dragonfly’ detected and identified mosquitoes with 82% confidence level in a real-time field trial. Moreover, the robot generated a map showing the distribution of mosquitoes. Overall, these advances highlight the potential of AI to automate mosquito identification, facilitating rapid interventions and real-time surveillance.

## IXODIDAE (TICKS)

Ticks are blood-sucking ectoparasites found in mammals, birds, reptiles and amphibians^28^. Identifying ticks is crucial for controlling diseases, such as spotted fever, Lyme disease, granulocytic anaplasmosis and babesiosis^29^. However, the traditional tick identification methods are time-consuming. With the advancement of CNNs, researchers have explored new approaches for automating tick identification with greater speed and efficiency. Our search yielded six articles from the Americas with accuracies ranging from 80-99% (Table 1).

Most studies on automated identification of ticks have been conducted in the United States. Justen et al.^30^ used the CNN TickIDNet model to identify three tick species, *Amblyomma americanum*, *Dermacentor variabilis* and *Ixodes scapularis*, by considering their stages of development (adult, nymph, or larva), sex, and feeding status. More than 12,000 images were analyzed and enlarged to 90,000 using data augmentation techniques. Images of ticks were captured from different backgrounds and angles. The overall accuracy was 87.8%, surpassing the identification accuracy of members of the public and health professionals, but falling short of that of entomology experts. This study highlights that the image quality, particularly the size of the tick in the photograph, significantly influences the accuracy of the model in identifying arthropods.

Omodior et al.^31^ compared the performance of the ResNet-50 model with a custom CNN to identify four tick species: *I. scapularis*, *D. variabilis*, *A. americanum,* and *Haemaphysalis* sp. This study used approximately 2,000 images, considering the sex and life stages of the ticks. Images were captured against different backgrounds, showing the dorsal and ventral positions. The customized CNN achieved 80% accuracy, surpassing that of the pretrained ResNet-50 model. Xu et al.^32^ developed the TickPhone app, a deep learning-based system for identifying ticks via smartphones using the LeNet network. The model was trained using more than 2,000 images, achieved 95.7% accuracy in identifying 31 species, including *I. scapularis*, *A. americanum*, and *D. variabilis*. This study emphasizes the importance of rapid identification for predicting the risk of tick-borne diseases. 

Akbarian et al.^33^ used a computer vision approach to differentiate *I. scapularis, A. americanum,* and *D. variabilis,* considering characteristics such as developmental stage, sex, and feeding status. This study involved capturing 12,588 images using smartphones. Additionally, 1,000 high-resolution tick images were captured using a camera mounted on a stereomicroscope to enhance the quality of the dataset. CNNs such as Inception-ResNet and knowledge-transfer techniques have been used to achieve 92% accuracy. Luo et al.^34^ tested five CNN models (VGG16, ResNet50, InceptionV3, DenseNet121, and MobileNetV2) to identify *A. americanum*, *D. variabilis* and *I. scapularis*. They used a set of 12,000 images from the TickReport passive tick surveillance program, which received samples from individuals, veterinary clinics, and partner agencies. The images were captured from multiple angles, including the dorsal and ventral angles, to assess the impact of perspective on model accuracy. Considering characteristics, such as developmental stage, sex, and feeding status, InceptionV3 model obtained the highest accuracy (99.5%).

**FIGURE 1:**
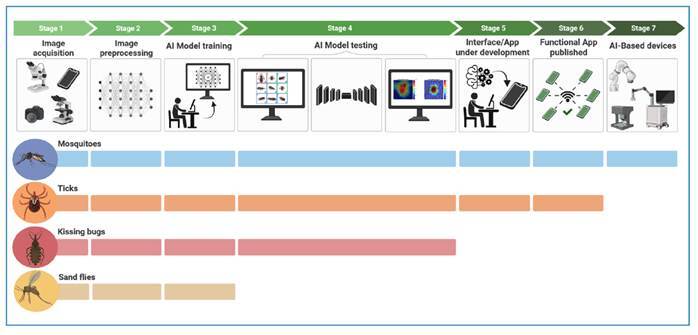
Advances in the development of automated identification systems for tropical disease vectors. The figure illustrates the seven stages of developing automated identification systems for four groups of vectors: mosquitoes, ticks, triatomines, and sand flies. The studies on mosquitoes and ticks are more advanced, with robust systems and AI-based devices already developed. Triatomine studies are close behind, with the prospect of developing applications within the next few years. Sand fly studies remain in the beginning stages.

In South America, Gomes et al.^35^ analyzed the ability of AlexNet, ResNet-50, and MobileNetV2 networks to identify vectors of spotted fever, such as *Amblyomma aureolatum*, *A. ovale*, and *A. sculptum*. This study organized a bank of 826 images, categorized according to sex, dorsal and ventral positions, and image resolution. The CNN models achieved accuracies of 94%, 92%, and 94% with sensitivities ranging from 59% to 100%, depending on the species, sex, position, and image resolution. In Asia, Akgül and Kaya^36^ used the YOLO-V3 network to detect ticks in a set of 1,500 images sourced from the Internet. These images were captured at various locations, such as skin and vegetation, at different resolutions. As the images were obtained from various online sources, they had no fixed positions. The model achieved an accuracy of 98.5%, demonstrating its efficacy for automatically identifying ticks in various environments. In Africa, Mudau et al.^37^ applied CNNs to quantify the parasitic load of ticks in cattle using infrared thermal imaging. The ConvNet and MobileNet models were trained using 1,124 thermographic images of animals captured at different angles, achieving training and validation accuracies of approximately 90% and 60% and 95% and 75%, respectively. Images of adult ticks (fed and unfed females and males) belonging to *Amblyomma hebraeum, Hyalomma marginatum, Rhipicephalus appendiculatus, Rhipicephalus (Boophilus)* spp., and *Rhipicephalus evertsi* were captured. This study highlights the potential of AI in monitoring tick infestations in livestock. Overall, these results indicated the high reliability of classifying important tick species using different algorithms (Table 1). In addition, some studies performed photo position tests, background variation tests, segmentation or Grad-CAM tests, and other applications. To date, no AI-driven robots have been developed to capture and identify ticks (Figure 1).

## TRIATOMINAE (KISSING BUGS)

The subfamily Triatominae comprises blood-sucking insects that transmit *Trypanosoma cruzi*, the etiological agent of Chagas’ disease^38^, which remains a major public health challenge. As triatomines continue to invade and infest human dwellings in the Americas^39^, developing technologies that can distinguish them from similar bugs is essential for improving Chagas disease vector surveillance^6^.

Our search revealed nine studies on the automated identification of triatomines in the Americas (Table 1). Gurgel-Gonçalves et al.^40^ first explored this approach, consistently achieving accuracies above 80%. They processed high-quality dorsal photographs taken with a specialized apparatus into landmarks and used ratios derived from these measurements to distinguish between 12 Mexican and 39 Brazilian triatomine species. Building on this, Khalighifar et al.^41^ employed the same image database alongside TensorFlow, an open-source software platform for deep learning algorithms, to improve identification rates. Using InceptionV3, they achieved identification rates of 83.0% and 86.7% for the Mexican and Brazilian species, respectively. When distributional data were incorporated, the identification rates increased markedly-to 95.8% for Mexican species and 98.9% for Brazilian species. Subsequent studies further improved accuracy by applying other algorithms to the same image bank of Gurgel-Gonçalves et al.^40^ and additional triatomine datasets, also yielding strong results^7^ ^,^ ^42^ ^-^ ^46^.

In later stages, the scope of automated triatomine identification broadened in both methodology and taxonomy. More complex deep learning algorithms, diverse datasets, and images of varying quality were incorporated. CNN-based models such as AlexNet and VGG16 outperformed classical statistical approaches, achieving accuracies between 93% and 100%, even for mobile phone images captured at different angles and resolutions^7^ ^,^ ^43^ ^,^ ^46^. Miranda et al.^7^ demonstrated that the AlexNet model can identify three genera (*Triatoma*, *Panstrongylus* or *Rhodnius*) with 100% accuracy, regardless of image orientation, while Miranda et al.^46^ reported 94.6% accuracy in identifying 48 vector species of medical importance. Similarly, Cochero et al.^45^ achieved 96% accuracy in classifying triatomines, using a CNN trained on mobile phone images, underscoring the feasibility of citizen-science-based systems. However, variations in performance across studies reflected differences in algorithms, image quality, number of taxonomic classes, and database curation.

Recent advances in the identification of triatomines have prompted Miranda and Gurgel-Gonçalves^47^ to propose a new challenge for automated identification systems: the need to distinguish triatomines from morphologically similar insects, such as phytophagous and predatory bugs, which are frequently misidentified as triatomines. To address this, they evaluated three CNNs (AlexNet, MobileNetV2, and ResNet-50) trained via transfer learning on a database of 707 dorsal bug images categorized by feeding habit. All models achieved over 94% accuracy, with ResNet-50 reaching 96.5% accuracy and perfect sensitivity and specificity for certain categories. Grad-CAM visualizations indicated that the models relied on different morphological features-such as the abdomen (connexivum) and hemelytra for blood-sucking bugs, and the head and pronotum for phytophagous and predatory bugs. These findings demonstrate that CNNs can accurately discriminate triatomines from other Heteroptera species even without lateral visualization of the mouthparts traditionally used by experts, marking a significant advance in automated entomological surveillance for both citizen science and fieldwork applications^47^.

To further advance automated triatomine identification, expanding the representativeness of image databases remains a crucial step. Miranda et al.^46^ showed that training AlexNet algorithm on a larger and more diverse dataset of 6,397 images from 65 species across seven genera and 27 countries markedly improved performance. The inclusion of images with varying resolutions, backgrounds, and lighting conditions enhanced model robustness against both morphological and photographic variability. The model achieved an overall accuracy of 92.8% across all species, rising to 94.6% when limited to the 48 most epidemiologically relevant species. The authors emphasized that incorporating species from diverse geographical regions and vectorial capacities is key to developing identification systems suitable for broad application. They also suggested that continuously expanding the database is fundamental to creating open-source tools that can be accessed via mobile devices and provide accurate real-time identification, thereby strengthening entomological surveillance programs throughout Latin America.

## PSYCHODIDAE (SAND FLIES)

Identifying phlebotomine sand flies is essential because they are vectors of several pathogens, such as *Leishmania* spp.^48^. Female sand flies transmit *Leishmania* species in a species-specific manner. Manual identification of internal structures is difficult; however, few studies have attempted to automate this process. We found four studies from the Old World. Fraiwan^49^ developed a deep learning AI system to identify three species (*Phlebotomus alexandri*, *Phlebotomus papatasi*, and *Phlebotomus sergenti)* in northern and central Jordan. Fraiwan et al.^50^ used images of the genitalia and pharynxes of sand flies captured in the field over a period of two years in Jordan and CNNs to identify them, achieving an accuracy of approximately 95%. Cannet et al.^51^ identified sand flies with an accuracy of over 77%, reaching 96% for American sand flies (Table 1). A new dataset was introduced to train and evaluate the recognition system for dipterans of medical and veterinary importance (Calliphoridae, Muscidae, Tabanidae, Ceratopogonidae, and Psychodidae). This system can identify dipterans based on wing morphology, thereby eliminating the need for internal organ inspection. This system can be implemented under both field and near-field conditions^52^.

Deep learning has shown promising results for the automated identification of cutaneous leishmaniasis lesion images^53^. Therefore, it is important to expand the leishmaniasis vector dataset to include neotropical phlebotomines and advance the development of application for sandfly identification based on image analysis. This application could help researchers and health professionals conduct surveillance of leishmaniasis vectors.

## CONCLUSIONS AND OUTLOOK

AI has revolutionized the identification and surveillance of disease vectors. Most studies have focused on applying AI to identify mosquito species, although the majority included fewer than ten species, except for two that analyzed 53 and 67 species. The vector species databases were generally limited in diversity, with the most representative group being the Triatominae, which included 65 species (41% of all described species). Approximately 30 algorithms have been used for automated vector identification, the most common being AlexNet, MobileNet, and ResNet. Moreover, these studies employed a wide range of hyperparameters.

Using CNNs for insect identification requires adaptations that extend beyond the original network architecture and typically involves three main stages. First, a suitable pretrained model must be selected (e.g., ResNet, EfficientNet, or MobileNet). Next, the convolutional layers that extract general features are frozen, and new classification layers are added. Finally, fine-tuning is conducted through hyperparameter optimization. Although most reviewed studies did not provide a comprehensive description of these steps, we summarized the algorithms and hyperparameters used in each case. Image resolutions varied both between and within studies. Several investigations have explored the impact of resolution, indicating that markedly low resolutions significantly reduce accuracy. However, in real-world scenarios, AI systems for vector identification cannot rely solely on high-resolution images. Identifying kissing bugs using low-resolution mobile phone images yielded similar accuracy to that obtained with high-resolution images. Comparable findings were reported for ticks, suggesting that resolution may not be a critical factor.

Most studies reported accuracy as the primary performance metric, with limited discussion of the implications of false positives and false negatives. A false positive (where a non-vector is misclassified as a vector) could trigger unnecessary public health alerts and resource expenditures, while a false negative (where a true vector is missed) could delay control interventions and facilitate pathogen transmission. Future studies should therefore incorporate a broader range of metrics, such as precision, recall, the F1 score, and the area under the curve (AUC).

Another important limitation across studies is the lack of systematic evaluation of how dataset characteristics influence performance. Factors such as database size, image resolution, background variability, and species diversity often have a greater impact on outcomes than the algorithm itself. These findings underscore the importance of dataset composition in determining model outcomes, and overlooking this factor can lead to overestimation of algorithmic performance. The results also depend heavily on hyperparameter settings, including architectural parameters (e.g., number of convolutional layers, filters, dropout rates, and activation functions) and training parameters (e.g., learning rate, optimizer, batch size, number of epochs, and regularization strategies). However, most reviewed studies did not report these configurations, limiting reproducibility. Some exceptions exist, such as Motta et al.¹⁵, who optimized hyperparameters for mosquito classification, and Miranda et al.⁴⁶, who evaluated multiple CNN architectures. Future research should systematically report hyperparameter choices in standardized tables to facilitate comparison across studies.

Most investigations remain in the algorithm training phase, with only one study in the Americas advancing to the development of applications or devices. Robots equipped with computer vision and CNNs, such as YOLOV4 have been used to capture mosquito images and identify species with high accuracy (Figure 1). Advancing this field requires expanding datasets to cover more species and regions, thereby enhancing identification systems. Significant progress has been made over the past seven years, highlighting the potential of AI in automated mosquito identification and contributing to rapid interventions and real-time surveillance.

Although encouraging advances have been made in automating tick identification, current studies primarily focus on tick species relevant to public health in North America. However, recent research has explored the application of AI to identify ticks in other regions where these ectoparasites play a key role in transmitting infectious agents. This review reinforces the need for further studies on spotted fever vectors in South America, which would strengthen epidemiological surveillance and support the development of deep learning tools for tick identification, benefiting both researchers and public health professionals in the region.

Following the inclusion of more triatomine species, automated identification systems could be made available through open-access applications (Figure 1). With an Internet-connected smartphone, users could photograph a triatomine, upload the image, and receive immediate identification and vector-related information. A key advancement in this direction is GeoVin (Geovin.com.ar), a platform that gathers geographic information on Argentinean triatomines via citizen reports of bug findings. Occurrence data (photos and geographic coordinates) online, stored, and automatically integrated into the GeoVin dataset. Applications based on automated visual identification systems may substantially strengthen Chagas disease vector surveillance while promoting citizen science.

Research on the automated identification of sand flies remains in its early stages (Figure 1). We found only four studies applying deep learning to sandfly identification. Several challenges remain, including expanding databases, testing optimal algorithms for this group, and determining the most informative morphological regions for males and females. Preliminary findings suggest that automated sandfly identification is promising. We also propose further studies on the automated identification of other vector groups, including snails, ceratopogonids, and simulids in the Americas. Our team is currently developing databases and planning future studies on these groups. We hope that technologies already applied to triatomines, culicids, and ticks can soon be extended to these additional vectors.

## Data Availability

Data-in-article: Research data is available in the body of the document (Table 1).
